# The Critical Shoulder Angle as a Prognostic Factor in the Arthroscopic Repair of Chronic Rotator Cuff Tears

**DOI:** 10.3390/jcm15124441

**Published:** 2026-06-09

**Authors:** Javier Álvarez de la Cruz, Marye Mercé Méndez Ojeda, Francisco Márquez Marfil, Nuria Álvarez Benito, José Luis Pais Brito

**Affiliations:** 1 Department of Orthopedic Surgery and Traumatology, University Hospital of the Canary Islands, 38320 San Cristóbal de La Laguna, Spain; fmarquezmarfil@gmail.com (F.M.M.); nuria.alvbe@gmail.com (N.Á.B.); jlpais@ull.edu.es (J.L.P.B.); 2 University of Health Sciences of La Laguna, 38200 San Cristóbal de La Laguna, Spain

**Keywords:** rotator cuff, critical shoulder angle, reintervention, pain

## Abstract

**Background**: Rotator cuff tears, a prevalent pathology with significant functional impact, primarily find their therapeutic approach in arthroscopic repair. The long-term success of this intervention is modulated by various factors, among which shoulder morphology stands out. In this context, the critical shoulder angle (CSA) has gained relevance as a potential predictor of postoperative prognosis. **Objectives**: To determine if the CSA influences the need for reoperation after retears and functional outcomes following the arthroscopic repair of chronic rotator cuff tears. **Methods**: A retrospective cohort study has been conducted, which includes 74 patients (between 47 and 86 years old, mean age of 58.85 ± 2.21 years) who underwent arthroscopic shoulder surgery for the repair of chronic rotator cuff tears by the traumatology service of a tertiary level hospital in the period between 2009 and 2022, to study variables such as the appearance of complications, reoperation, and functional outcomes. **Results**: Patients with a CSA greater than 40° represent 56.75% (n = 42). Functional improvement was achieved in 59.52%, in contrast to 40.48% of patients who did not obtain significant functional improvement (OR = 1.470; 95% CI = 1.009–2.141). The reoperation rate in this group was 21.4% (n = 9) (OR = 1.562; 95% CI = 1.085–2.249). **Conclusions**: This study provides statistically significant evidence that a high CSA (>40°) behaves as a risk factor for the reoperation rate and poorer functional outcomes after the arthroscopic repair of chronic rotator cuff tears.

## 1. Introduction

Rotator cuff (RC) tear is a prevalent pathology, which can range from 9% in patients under 20 years to more than 60% in patients over 80 years [1]. This pathology generates pain and functional limitation of the shoulder, with arthroscopic repair being the most common treatment option. However, its long-term success depends on various factors [2], including shoulder morphology, where the critical shoulder angle (CSA) has gained relevance for its potential influence on the postoperative prognosis.

The CSA is defined as the angle formed by the intersection of two lines: one that extends from the lateral edge of the acromion to the center of the humeral head, and another parallel to the inferior border of the glenoid. A high CSA (>38°) is associated with a reduced subacromial space [3], consequently generating an increase in the pressures to which the rotator cuff is subjected (especially during abduction and external rotation of the shoulder) and, therefore, accelerates its degeneration and increases the risk of tears [4]. Compared to the asymptomatic population, patients with RC tears have higher CSA values [5]. Furthermore, the severity of RC involvement increases as CSA values increase [6].

While some studies report a higher risk of retear of the sutured tendon in patients with a high CSA [7,8], others find no association between this angle and long-term functional outcomes and even suggest that the CSA should not be used as a clinical predictor to evaluate the risk of a new rotator cuff tear after arthroscopic surgery [9,10,11]. This disparity in evidence, attributable to methodological heterogeneity, population variability, and confounding variables, generates considerable controversy. Studies differ in the definition of the CSA, measurement techniques, inclusion criteria, and evaluation of results. Some authors claim that patient activities over several decades could induce not only cuff lesions but also bone remodeling at the acromial level [12], contributing to the disparity in results. It is also necessary to describe the type of tear and repair techniques that vary between studies, making direct comparison difficult.

Finally, it should be highlighted that the prevalence of these tears increases with age [13], and is also influenced by sex (prevalence is higher in males) [13], the patient’s comorbidities (diabetes and obesity [2]), and other habits such as smoking [14], all of which can influence postoperative functionality independently of the CSA.

Studies differ in the definition of the CSA, measurement techniques, inclusion criteria, and evaluation of results. Some authors claim that patient activities over several decades could induce not only cuff lesions but also bone remodeling at the acromial level [4]. Finally, it should be highlighted that the prevalence of these tears increases with age, and is also influenced by sex, comorbidities, and habits [1,2]. Despite the growing interest in the CSA, significant gaps remain in the literature regarding its precise impact on surgical success, largely due to this methodological heterogeneity. Therefore, this study aims to address these gaps by answering the following specific research questions: (1) Does a high CSA (>35° or >40°) significantly increase the risk of reoperation following arthroscopic repair of chronic rotator cuff tears? and (2) How does the CSA influence long-term functional outcomes and residual pain after arthroscopic suture?

## 2. Materials and Methods

### 2.1. Study Design and Participant Selection

A retrospective cohort study was conducted to evaluate patients with a chronic rotator cuff tear who underwent arthroscopic repair. The intervention period comprised surgeries performed by the traumatology service of a tertiary level hospital between 2009 and 2022. A purposive, consecutive sampling technique was employed, wherein all patients who met the eligibility criteria during the designated timeframe were initially selected to minimize selection bias. Inclusion criteria strictly required patients to have an isolated supraspinatus tear or a tear concomitant with other rotator cuff tendons, diagnosed by ultrasound or magnetic resonance imaging, with no prior intervention on the affected shoulder. Furthermore, a true anteroposterior plain radiograph of the operated shoulder was mandatory to accurately perform the CSA measurement. Patients were excluded if they declined verbal consent via a phone call, or if previous plain shoulder radiographs were unavailable or unmeasurable. To isolate the variables, patients with subacromial impingement diagnosed by ultrasound or MRI (involving radiological bone or ligamentous factors such as Bigliani type III Acromion, persistent os acromiale, or multi-bundle coracoacromial ligaments) were excluded. Additional exclusion criteria encompassed the presence of concomitant shoulder pathologies requiring surgical modification (e.g., subscapularis or labral tears), psychiatric disorders, and reoperations solely for postoperative stiffness.

### 2.2. Data Collection and Study Variables

The study was approved by the research ethics committee of the Complejo Hospitalario Universitario de Canarias (protocol code: CHUC_2024_10, with approval date of the ethics Hospital committee on 29 February 2024). Eligible patients were registered using an anonymized coding system (e.g., PAC-001), and demographic and clinical data were extracted from the computerized clinical records of the SAP system. Evaluated variables included age, sex, smoking status, hand dominance, and the operated side. The CSA was defined and measured in degrees as the angle formed by the intersection of a line extending from the lateral edge of the acromion to the center of the humeral head, and another parallel to the inferior border of the glenoid. Clinical outcomes were gathered via patient interviews, focusing on residual pain, functional changes post-surgery, and a subjective percentage-based comparison of functionality against the contralateral healthy shoulder. Patient satisfaction was quantified by their willingness to undergo the surgery again if necessary.

### 2.3. Statistical Data Analysis and Sample Size Justification

A formal sample size calculation was performed to ensure robust statistical power. Based on previous evidence and expert consensus, a 20% difference in the reoperation rate between patients with high and normal CSA was expected. Assuming a 10% variability in the reoperation rate, setting the significance level at 0.05, and targeting a statistical power of 80%, it was determined that a cohort of 74 patients was optimal and viable for achieving reasonable statistical power. Data analysis was conducted using Microsoft Excel and SPSS V.30. Cross-tabulations were used to examine variable relationships. While multivariable regression models were initially considered to control for potential confounders, the strict sample size of 74 patients limited the statistical power for complex multivariable analyses; therefore, rigorous inclusion and exclusion criteria were proactively applied to ensure sample homogeneity and control confounding variables pre-analytically. Survival analysis regarding the reoperation rate across different CSA values was modeled using the Kaplan–Meier test. The rationale for the specific CSA cutoff points was twofold: 35° was established based on existing literature identifying it as a traditional diagnostic marker for tear presence, while the 40° threshold was justified as a more sensitive, specific prognostic marker for clinical failure, as suggested by contemporary outcome studies.

## 3. Results

The study included a total of 74 patients, 48 women (64.86%) and 26 men (35.13%). The mean age at the time of the intervention was 58.85 ± 2.21 years, with the group aged 60 to 69 being the most prominent, representing 41.89% of the patients studied (n = 31). The most frequently operated side was the right with 72.97% (n = 54). Of the total patients, 14.86% (n = 11) had to be re-operated on. Key epidemiological variables are summarized in Table 1.

The presence of residual pain after surgery was observed in 58.1% (n = 43) of the patients. However, 81% (n = 60) experienced a significant improvement in postoperative pain compared to the pain before the intervention. Regarding shoulder functionality, an improvement was recorded after surgery in 67.5% (n = 50) of the cases, while 32.4% (n = 24) maintained equal or lower functionality. In addition, 82.4% (n = 61) of the patients were satisfied with the results of the operation and with the management of the procedure by the hospital service, indicating their willingness to undergo the intervention once more, if necessary.

The CSAs of the group of patients studied are ordered as follows. Patients with angles less than 35° account for 12.16% (n = 9), followed by angles between 35–40° with 31.1% (n = 23), thirdly between 40–45° being the most numerous group with 39.2% of the sample (n = 29) and, finally, angles greater than 45° which account for 17.6% (n = 13). To observe whether the critical shoulder angle influences the risk of reoperation of the arthroscopic repair after RC tear and the functional results, a group division has been made with two cutoff points. The first cut is made at 35°, making a division between patients with angles greater than 35° and patients with angles less than 35°. The second cut is made at 40° following the same methodology as the previous one.

### 3.1. Cutoff Point at 35°

Regarding patients with a CSA of less than 35°, they constitute 12.16% of the sample (N = 9). Regarding functional results, 6 of the patients showed postoperative improvement, compared to 3 who obtained worse or equal functional results (OR = 0.980; 95% CI = 0.267–3.594). In addition, 1 needed reoperation (11.11%) (OR = 0.716; 95% CI = 0.099–5.173). Of the 65 patients with an angle greater than 35°, 44 found functional improvement (67.69%), which contrasts with the 21 patients (32.32%) with worse or equal results after surgery (OR = 1.003: 95% CI = 0.839–1.199). 10 required reoperation (15.38%) (OR = 1.041; 95% CI = 0.845–1.284).

### 3.2. Cutoff Point at 40°

Patients with a CSA of less than 40° account for 43.24% (N = 32). An improvement in functionality was achieved in 78.12% of these patients (N = 25), compared to 7 (21.88%) with worse or equal functionality results (OR = 0.549; 95% CI = 0.276–1.090). On the other hand, 6.25% (n = 2) required reoperation (OR = 0.382; 95% CI = 0.106–1.373). As for patients with CSA greater than 40°, they account for 56.75% (n = 42). Functional improvement was achieved in 59.52% (N = 25), in contrast to 40.48% of the patients (N = 17) who did not obtain significant functional improvement (OR = 1.470; 95% CI = 1.009–2.141). Reoperation rate of 21.4% (n = 9) (OR = 1.562; 95% CI = 1.085–2.249).

**Figure 1:**
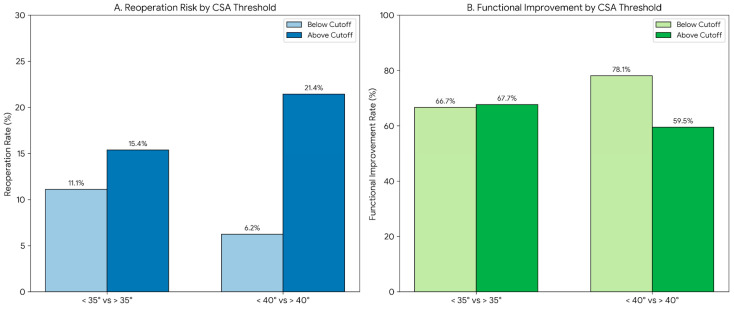
Comparative Analysis of Clinical Outcomes by CSA Thresholds. Graph (**A**) details the reoperation rates, comparing the impact of the 35° and 40° cutoff points. It is observed that the 40° threshold offers a much clearer prognostic differentiation: while the difference at 35° is less pronounced (15.4% vs 11.1%), the group with a CSA > 40° presents a significantly higher reoperation rate (21.4%) compared to the < 40° group (6.2%). Graph (**B**) shows the postoperative functional improvement rates. Patients with a CSA < 40° achieve a functional success rate of 78.1%, compared to 59.5% for those with angles exceeding 40°. These data visually identify the 40° threshold as a more sensitive and specific predictor of surgical success and repair stability than the traditional 35° cutoff.

**Figure 2:**
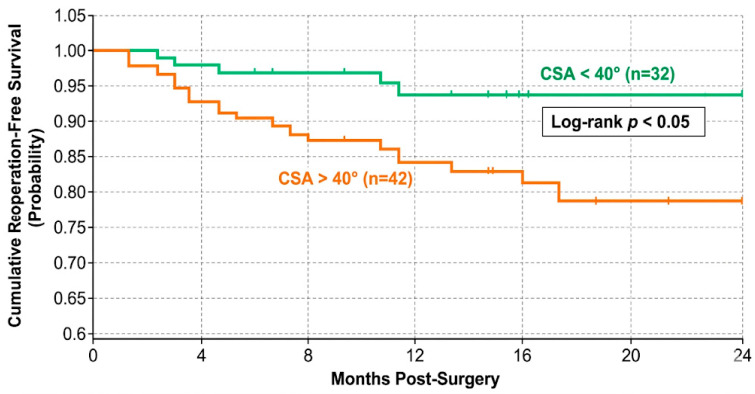
This Kaplan- Meier curve illustrates the cumulative probability of remaining free from reoperation over a 24-month follow-up period following arthroscopic repair. The green line represents patients with a CSA < 40°, who show superior clinical stability over time. Conversely, the orange line (patients with CSA > 40°) shows an early and progressive decline in clinical survival, reflecting a higher rate of suture failure and the need for reintervention. The significant divergence between the two curves, validated by the Log-rank test (*p* < 0.05), statistically confirms that an angle greater than 40° is a critical risk factor for medium-term clinical failure.

As a visual summary of the documented clinical findings, a detailed comparison of the reoperation rates and functional improvement percentages across the various CSA thresholds is illustrated in Figure 1. Additionally, the temporal distribution of these reoperations and the cumulative probability of clinical survival throughout the 24-month follow-up period are presented in the Kaplan-Meier survival analysis curve in Figure 2.

## 4. Discussion

This study has investigated the influence of the critical shoulder angle on reoperation and functional outcomes after arthroscopic repair of the rotator cuff. Our findings reveal a statistically significant association between a CSA greater than 40 degrees and a higher risk of reoperation, as well as poorer functional outcomes in operated patients.

Several mechanisms are proposed to explain the relationship between an increased CSA and poorer outcomes in repair. First, a reduced subacromial space can compress the rotator cuff tendon, increasing the risk of wear and tear. In addition, these pressure increases could hinder the blood supply to the injured areas, so a high CSA could also hinder proper healing of the tendon after repair.

Acromial anatomy seems to play an important role in both the onset and prognosis of this pathology. Pandey et al. (2016) demonstrated how scapular morphology, including the CSA, affects the integrity of the rotator cuff [15]. They describe how the presence of a spur on the acromion is closely related to a full-thickness rotator cuff tear, but not to partial tears. However, the type of acromion seems not to be related to the rotator cuff tear. Moor et al. (2014) established a relationship between individual scapular anatomy, which includes the CSA, and rotator cuff tear [5]. It was hypothesized that a large acromial cover with an upwardly inclined glenoid fossa would be associated with degenerative rotator cuff tears and, conversely, that a short acromion with a downwardly inclined glenoid would be associated with glenohumeral osteoarthritis. Furthermore, Lin et al. (2020) demonstrated a positive correlation between the CSA and the size of the rotator cuff tear, suggesting that a reduced subacromial space could predispose to more extensive injuries [16].

Regarding the influence of the CSA after arthroscopic repair of the RC, Li et al. (2021) determined that a CSA > 38° is associated with a higher risk of RC tendon tear after repair [8]. However, the CSA does not seem to influence the functional outcomes of the operated patients. In the same vein, Lapner et al. (2022) reported that a CSA > 40° was associated with a higher risk of tear, but without obtaining functional differences at 2 years of follow-up [17]. Liu et al. (2023) also describe how despite the increased risk of re-tear in patients with high CSA, the functional results do not differ [7].

Some studies do not find a clinically relevant association between the CSA and rotator cuff tears. Spiegl et al. (2016) describe how it correlates better with osteoarthritis than with rotator cuff tears, being more accurate on radiographs than on magnetic resonance imaging [18]. Bjarnison et al. (2017) found no relationship between the CSA and RC tears, but they did with glenohumeral arthrosis, with an OR of 2.25 of developing arthrosis if the patient had a CSA lower than 30° [19]. Unlike this study, the previously described ones do not find significant differences in terms of functionality. Therefore, they do not support the practice of performing a lateral acromioplasty to reduce the CSA, as it could increase the risk of developing OA without decreasing the risk of developing future cuff tears [19].

The standardized quantification of post-operative functional optimization and upper-extremity kinematics relies heavily on validated, multidimensional clinimetric instruments. Universally recognized frameworks—such as the Constant-Murley score, the American Shoulder and Elbow Surgeons (ASES) index, and the Disabilities of the Arm, Shoulder, and Hand (DASH) outcome measure—serve as the clinical benchmarks for cross-study comparison, systematically evaluating discrete parameters of pain, range of motion, and patient-reported quality of life [20,21,22]. However, the unique geographical fragmentation and distinct demographic distribution characterizing our specific island territory present substantial logistical barriers to executing standardized, in-person clinical reviews for the entirety of the cohort. Consequently, to proactively navigate these territorial constraints and prevent critical patient attrition during long-term follow-up, an adapted, structured telephonic assessment protocol was implemented as a pragmatic strategy to ensure comprehensive data capture while preserving the statistical power of the study.

The practical and clinical implications of these findings are highly relevant for modern arthroscopic practice. Our results clearly articulate that a high CSA (>40°) must be considered a decisive risk factor for clinical failure. This evidence mandates the inclusion of preoperative radiographic CSA assessment as an indispensable tool for precision surgical decision-making and planning. For patients identified with extreme scapular morphology, surgeons should alter their management protocols; this could include utilizing enhanced biological augmentation techniques during repair, modifying post-operative rehabilitation to be more conservative, and conducting realistic patient counseling regarding the elevated risk of retear and suboptimal functional recovery. Furthermore, the identification of a high CSA inevitably raises the debate regarding the potential benefit of performing a concurrent lateral acromioplasty. While some historical literature cautions against this due to the theoretical risk of accelerating osteoarthritis without clear retear prevention [19], recent investigations suggest that surgically correcting the CSA to near 35° via 2D-planned lateral acromioplasty can effectively normalize the deltoid force vector. As demonstrated by Toro et al. (2024) [23], lateral acromioplasty specifically aims to reduce the excessive superior shear forces on the repaired tendon rather than acting merely as a subacromial decompression. However, whether this radiographic correction reliably translates into improved functional scores and a sustained reduction in retear rates across diverse patient demographics remains a topic of active surgical research [23]. Consequently, until a universal consensus is reached, the decision to surgically modify an extreme CSA must be highly individualized, carefully balancing the biomechanical benefits against the risks of altering the native coracoacromial arch.

Considering the current controversy, it is crucial to interpret the present evidence with caution. A definitive causal relationship between the CSA and the risk of rotator cuff reoperation cannot be established. In addition, the CSA must be evaluated on an individualized basis, considering other factors such as age, sex, type of tear, and repair technique. Consequently, it would be relevant for the future to conduct studies with larger, prospective, and randomized sample sizes, also needing to homogenize definitions, measurement techniques, and evaluation criteria to obtain comparable results.

### Limitations

This study has several methodological constraints that must be critically evaluated. First, the retrospective design and the relatively small sample size of 74 patients limit the ability to establish a definitive, unquestionable causal relationship between the CSA and the risk of rotator cuff reoperation. Although the sample size was rigorously justified through a power analysis, the cohort size restricted the use of advanced multivariable regression models, which could have provided deeper control over nuanced confounders. Second, patients were operated on over a wide timeframe (2009–2022), introducing a highly variable follow-up period and potential variations in surgical hardware or techniques over the decade. Finally, due to territorial and demographic constraints that make in-person follow-ups complex for our specific population, functional assessments were conducted via telephone interviews. While this methodology prevented significant patient loss, reliance on objective, simplified questioning rather than validated, comprehensive clinical scores (such as Constant, ASES, or DASH) may limit the standard generalizability of the functional outcomes reported. Consequently, future research should focus on prospective, randomized trials with larger cohorts to homogenize these evaluation criteria and further validate these findings.

## 5. Conclusions

This study provides statistically significant evidence that a high critical shoulder angle (>40°) is a decisive risk factor for clinical failure and poorer functional outcomes following the arthroscopic repair of chronic rotator cuff tears. While the traditional 35° threshold remains a valuable diagnostic marker for the presence of a tear, the 40° cutoff offers superior prognostic sensitivity for identifying patients at high risk of reoperation. These findings underscore the necessity of preoperative radiographic assessment as a tool for precision surgical planning. For patients with extreme scapular morphology, surgeons must adopt a personalized management strategy that includes enhanced biological augmentation, modified rehabilitation protocols, and realistic patient counseling. Future research should focus on prospective, randomized trials to determine whether the surgical reduction in extreme CSAs through aggressive lateral acromioplasty consistently improves long-term clinical and structural success.

## Figures and Tables

**Table 1:** Summary of baseline demographic characteristics and key epidemiological variables of the study cohort (*n* = 74), including the distribution of sex, smoking status, hand dominance, and surgical side, stratified by age group.

Characteristic	Total (*n* = 74)	Age Group: 18–32 Years (*n* = 4)	Age Group: 33–47 Years (*n* = 6)	Age Group: 48–62 Years (*n* = 25)	Age Group: 63–77 Years (*n* = 27)	Age Group: 78+ Years (*n* = 12)
**Sex**						
Male, n (%)	26 (35.1%)	2 (50.0%)	3 (50.0%)	9 (36.0%)	9 (33.3%)	3 (25.0%)
Female, n (%)	48 (64.9%)	2 (50.0%)	3 (50.0%)	16 (64.0%)	18 (66.7%)	9 (75.0%)
**Smoking Status**						
Smoker, n (%)	21 (28.4%)	1 (25.0%)	2 (33.3%)	7 (28.0%)	8 (29.6%)	3 (25.0%)
Non-smoker, n (%)	53 (71.6%)	3 (75.0%)	4 (66.7%)	18 (72.0%)	19 (70.4%)	9 (75.0%)
**Hand Dominance**						
Right-handed, n (%)	54 (73.0%)	3 (75.0%)	5 (83.3%)	18 (72.0%)	19 (70.4%)	9 (75.0%)
Left-handed, n (%)	20 (27.0%)	1 (25.0%)	1 (16.7%)	7 (28.0%)	8 (29.6%)	3 (25.0%)
**Surgical Side**						
Right side, n (%)	54 (73.0%)	3 (75.0%)	5 (83.3%)	18 (72.0%)	19 (70.4%)	9 (75.0%)
Left side, n (%)	20 (27.0%)	1 (25.0%)	1 (16.7%)	7 (28.0%)	8 (29.6%)	3 (25.0%)

## Data Availability

The original data presented in the study are openly available in Figshare at https://doi.org/10.6084/m9.figshare.31989972 (accessed on 12 April 2026).
